# Online Fully Automated System for Hydrogen/Deuterium-Exchange Mass Spectrometry with Millisecond Time Resolution

**DOI:** 10.1021/acs.analchem.2c05310

**Published:** 2023-03-10

**Authors:** Monika Kish, Victoria Smith, Natasha Lethbridge, Lindsay Cole, Nicholas. J. Bond, Jonathan J. Phillips

**Affiliations:** † Living Systems Institute, Department of Biosciences, University of Exeter, Stocker Road, Exeter, EX4 4QD, U.K.; ‡ CPI, Darlington DL1 1GL, U.K.; § Applied Photophysics Ltd, Leatherhead KT227BA, U.K.; ∥ Analytical Sciences, Biopharmaceutical Development, BioPharmaceuticals R&D, AstraZeneca, Milstein Building, Granta Park, Cambridge CB21 6GH, U.K.; ⊥ Alan Turing Institute, British Library, London NW1 2DB, U.K.

## Abstract

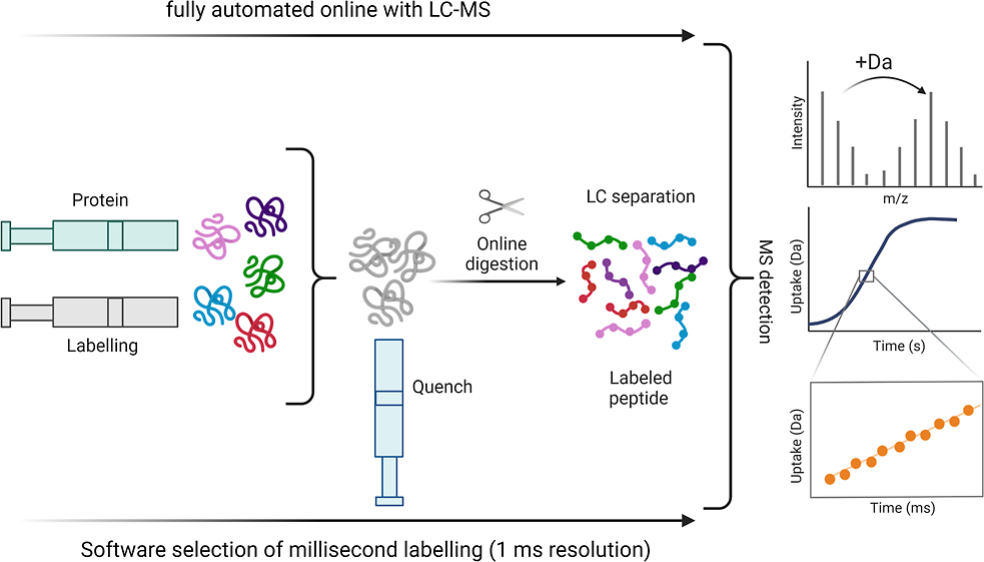

Amide hydrogen/deuterium-exchange mass spectrometry (HDX-MS) is a powerful tool for analyzing the conformational dynamics of proteins in a solution. Current conventional methods have a measurement limit starting from several seconds and are solely reliant on the speed of manual pipetting or a liquid handling robot. Weakly protected regions of polypeptides, such as in short peptides, exposed loops and intrinsically disordered the protein exchange on the millisecond timescale. Typical HDX methods often cannot resolve the structural dynamics and stability in these cases. Numerous academic laboratories have demonstrated the considerable utility of acquiring HDX-MS data in the sub-second regimes. Here, we describe the development of a fully automated HDX-MS apparatus to resolve amide exchange on the millisecond timescale. Like conventional systems, this instrument boasts automated sample injection with software selection of labeling times, online flow mixing and quenching, while being fully integrated with a liquid chromatography–MS system for existing standard “bottom-up” workflows. HDX-MS’s rapid exchange kinetics of several peptides demonstrate the repeatability, reproducibility, back-exchange, and mixing kinetics achieved with the system. Comparably, peptide coverage of 96.4% with 273 peptides was achieved, supporting the equivalence of the system to standard robotics. Additionally, time windows of 50 ms–300 s allowed full kinetic transitions to be observed for many amide groups; especially important are short time points (50–150 ms) for regions that are likely highly dynamic and solvent- exposed. We demonstrate that information on structural dynamics and stability can be measured for stretches of weakly stable polypeptides in small peptides and in local regions of a large enzyme, glycogen phosphorylase.

## Introduction

Understanding the relationship between the protein structure and biological function is essential to our knowledge of protein systems and to the design of novel drug therapies. One of the few methods capable of resolving local perturbations in stability down to the amino acid level^1^ in proteins is hydrogen/deuterium-exchange mass spectrometry (HDX-MS).^2−6^ Over the past decade, the use of HDX to explore protein structure–function relationships has rapidly increased. It can be used to answer questions about epitope mapping,^7^ protein–drug binding,^8^ protein–protein interactions,^9^ aggregation,^10^ and allosteric regulation.^11^ This is largely due to the commercial availability of a fully automated system for HDX-MS.^12^

In a conventional HDX-MS protocol, the protein is exposed to high levels of deuterium oxide (D_2_O) for different periods of time.^13^ Labile backbone amide hydrogens are exchanged with deuterium, where the rate at which each residue exchanges to deuterium is directly related to the protein structure, more so to the hydrogen network and solvent assessable area.^14^ After digestion and separation, the mass of each peptide and its mass shift are determined by MS.^15^ Since 2001, many challenges and shortcomings of HDX have been met, including automated sample preparation, automated data processing,^16,17^ improved digestion,^18^ liquid chromatography (LC),^12^ sensitivity of the MS, and, overall, the spatial resolution.^1,19,20^ Several automation systems have been reported so far.^13,21,22^ LEAP Technologies^21^ commercialized a system called the “H/D Platform”. This includes a CTC/PAL-based sample preparation and autosampler system, a chamber for digestion, as well as their own sample preparation and sample injection sequencing software. However, the commercial CTC/PAL-based front-end sample preparation systems from LEAP are only suitable for reproducible deuterium incubation times in the range of seconds or longer.^23^ There is growing interest to perform HDX at the sub-second timescale to measure the structural dynamics and stability of classes of molecule that are currently intractable (e.g., peptide hormones, neurotransmitters,^24^ and intrinsically disordered proteins/regions^6^). A fast HDX-MS automated system is a specific sample prep solution that covers the missing milliseconds to seconds range. A small number of academic laboratories have reported experimental systems and approaches to obtain millisecond HDX-MS data: microfluidic chips,^25−31^ a quench flow setup,^32−34^ an online quench flow setup,^2,35^ a capillary mixer,^36−38^ native MS coupled with HDX,^39^ and a pH modulation method.^40^ These allow the sub-second HDX-MS analysis of unstructured or highly dynamic solvent-exposed regions of biological structures and are applicable to a wide range of protein molecules. As such, millisecond HDX-MS can be used to provide further insights into epitope mapping, protein–drug binding, protein–protein interactions, aggregation, and effects of mutation on protein conformation and dynamics, especially when it comes to fast-exchanging structures. None of the previously developed systems offers a fully automated, online LC–MS solution for HDX-MS with software selection of mixing times at a single millisecond time resolution.

Here, we describe the construction, validation, and implementation of an online flow rapid mixing and quenching HDX system. With this system, we achieved reproducible and repeatable HDX of 50 ms to 5 min on unstructured peptides and a large enzyme, attainable at a time resolution of 1 ms. To demonstrate the capability of this instrument to perform standard “bottom up” workflows, we analyzed the large protein glycogen phosphorylase b (GlyPb) dimer (195 kDa), commonly used as a standard.^41^ We achieved digestion, coverage, and kinetics comparable to those of standard conventional HDX systems in a fully automated manner, including software-controlled sample handling, mixing, and analysis. Furthermore, we demonstrated the broad applicability of such a system by measuring rapid exchange kinetics of a large protein on the millisecond timescale with 273 peptides, corresponding to 96.4% protein coverage.

## Experimental Section

### Chemicals and Reagents

Chemicals were purchased as follows: potassium phosphate dibasic (99.9%), potassium phosphate monobasic (99.9%), tris hydrochloride, tris(2-carboxyethyl)phosphine hydrochloride (TCEP), and dimethyl sulfoxide-d6 (99.96%) from Sigma; dimethyl sulfoxide from Fisher Bioreagents; deuterium oxide (99.9% D) from Goss Scientific; and water (H_2_O), acetonitrile (ACN), and formic acid (99.5%) Optima LC/MS Grade were from Fisher Scientific. All other ultrapure water used was purified on a Milli-*Q* Advantage A10 system (Merck). Five peptides were used to prepare the peptide mixture, including bradykinin (RPPGFSPFR) obtained from Sigma, leucine enkephalin (YGGFL) from Waters, CN-AFP (DTASDAAAAAALTAANAAAAAEKTAADAAAAAAATAA) from Peptide Synthetics, and cTPRH1 (AEAWYNLGNAYYK) and cTPRS (AEAKQNLGNAKQK) synthesized in-house on a Biotage parallel synthesizer (SYRO II) on wang resin with purification by reverse phase against a C8 column (Polaris). GlyPb from rabbit muscle was purchased from Sigma.

### Sample Preparation

All peptides were dissolved in DMSO or DMSO-*d*_6_ for the determination of the maximum deuteration level and back exchange of the system. The peptide mixture was prepared with each peptide at a concentration of ≈5 μM in 20 mM potassium phosphate buffer at pH 7.40. GlyPb was dissolved and diluted in 40 mM tris hydrochloride and 1 mM TCEP at pH 7.00 to a final concentration of 10 μM.

### “ms2min” Design

**Figure 1:**
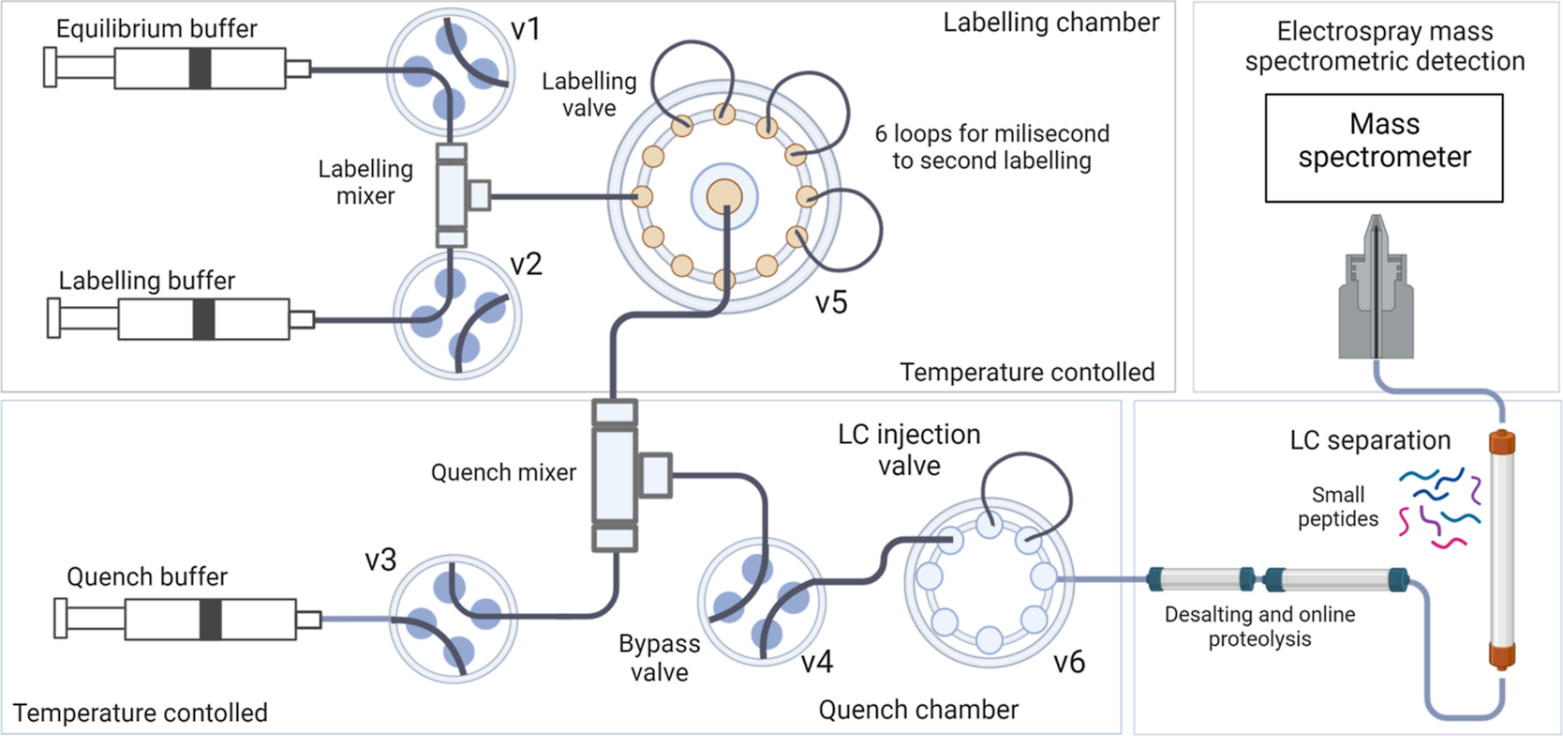
Characterization of the fully automated ms2min system. Syringes are used for storing/delivering the protonated, deuterated, and quenching buffers. Delivery/fill are automated through valves V1-3. Labeling is achieved by coordinating the fluid velocity and the length of the chosen delay loop prior to mixing with the quench in the quench mixer. The quenched labeled “sample” is then introduced into the LC–MS system.

The millisecond HDX (ms2min) system was designed to allow fully automated HDX labeling from the low millisecond to minutes timescale, with high temporal resolution, temperature control, and online connection to a two-dimensional chromatography system for conventional “bottom-up” workflows. A schematic representation of the system is shown in Figure 1. Briefly, three syringes (equilibrium, labeling, and quench) store and deliver the buffers (protonated, deuterated, and quenching buffers, respectively), with automated fill/delivery via valves V1–3. The unlabeled sample is introduced through V1 in the flow tube before the labeling mixer. The labeling time is varied by matching the fluid velocity and the choice of delay loop volume from six installed loops on labeling loop valve V5 (only three loops are shown in Figure 1 for clarity) prior to mixing with the quench in the quench mixer (QM). During the labeling reaction, the flow is directed to waste by a bypass valve (V4) to allow high flow rates. In a subsequent step, the quenched labeled sample is loaded onto the high-pressure LC injection valve V6 and introduced into the LC system, enabling automated online LC–MS for “bottom-up” workflows.

### Calibration of the Mixing Times and Time Accuracy

Time point accuracy is determined by relating the applied velocities of the labeling and equilibrium buffer syringes (stepper motors) with the accurate calibration of the volume of each of six delay loops between the labeling D_2_O mixer (DM) and QM. Each syringe stepper motor’s step to volume was determined gravimetrically by measuring the volume of H_2_O flushed through the system, with flow-rate accuracy determined to be within 1% for all syringes. The delay loops were then calibrated using 24 mg/L nicotinic acid (Sigma-Aldrich) in 0.1 M HCl loaded onto the equilibrium syringe; the quench and label buffer syringes were loaded with 0.1 M HCl. 1 mL of nicotinic acid was delivered from the equilibrium syringe for delay loop volumes under a nominal 300 μL and 2 mL delivered for larger delay loop volumes to ensure full loading of the delay loop. Next, 1 mL of HCl was delivered from the quench buffer syringe, flushing the final tubing section. Finally, 1 mL of HCl from the label buffer syringe was collected, and the loop volume was calculated by the dilution of nicotinic acid from the equation below
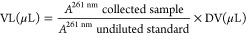
1where VL is the volume of the loop and DV is the delivered volume, which is equal to 1000 μL.

### Achieving Turbulent Flow for Mixing at All Time Points

Achieving turbulent flow for mixing at all time points is fundamental to the design of the quench flow system presented here. Therefore, the simple measurement of loop volume is sufficient to achieve accurate labeling times. The different loop volumes of the labeling valves allow the maintenance of high flow rates that achieve turbulent flow through the mixers while allowing progressively longer labeling times. To ensure fast mixing, it is important to achieve turbulent flow through all mixers; this is achieved at Reynolds numbers more than 2000. The Reynolds number R can be calculated from the following equation.
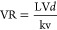
2where LV is the linear velocity of the flowing liquid in mm s^–1^, *d* is the diameter of the tubing in mm (0.733 mm for both the labeling mixer and the QM), and kv is the kinematic viscosity of the liquid (1 mm^2^ s^–1^ for water at 20 °C).

Flow rate *F* in μL s^–1^ can be converted to LV below.
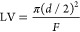
3

The software has configurable system settings for maximum and minimum total flow rates. The maximum flow rate was determined empirically by running progressively faster flow rates until we experienced a drive syringe stall and then subtracted a 10% safety margin from the flow rate. This maximum total flow rate was 2700 μL s^–1^ through the labeling mixer and 5400 μL/s through the QM. We calculated a Reynolds number of 4696 through the labeling mixer and 9372 through the QM (given a standard 1:1 labeled sample vs quench mixing ratio). This is well into the turbulent mixing region. The minimum total flow rate was set to 1154 μL s^–1^ through the labeling mixer, providing a minimum Reynolds number of 2000 and ensuring turbulent mixing.

The delay loops of the labeling valve volumes were made to provide a 1.5- to 2-fold increase in volume compared to the previous loop, ensuring no gaps in possible labeling times.

At sufficiently long time points (over ∼500 ms), a second strategy is to first label the sample through the labeling mixer into a delay loop of sufficient volume to contain all labeling sample and then stop the flow. After a delay, the labeling sample is pushed out and mixed with the quench in the quenching mixer. The flow through the labeling mixer is carried out at a preset flow rate of 2400 μL s^–1^, providing a Reynolds number of 4165, and the flow through the QM is at a flow rate of 4800 μL s^–1^ to provide a Reynolds number of 8330.

Timing accuracy was determined using the base catalyzed hydrolysis of 2,4-dinitrophenyl acetate (DNPA, Acros Organics) to 2,4-dinitrophenol (DNP).^42^ A solution of 2 mM DNPA in 2% (*v*/*v*) DMSO and 0.1 M HCl (equilibrium syringe) was mixed 1:10 with 0.5 M NaOH (labeling buffer syringe) and quenched 1:1 with 1 M HCl (quench buffer syringe). DNPA and DNP in the acid quenched reaction solutions were separated on a C18 reverse phase column (Telos) with isocratic 50% acetonitrile/water and 0.1% trifluoroacetic acid and were detected by UV absorbance (254 nm). Time points between 40 and 500 ms were collected in triplicate, and 0 ms time points of unreacted solutions where acid was added by hand before the NaOH was added. For direct comparison, the same reaction solutions were analyzed using UV absorbance at 360 nm (1–500 ms) in a SX20 stopped-flow spectrophotometer (Applied Photophysics Ltd). Both ms2min and stopped-flow data sets were fitted with a single exponential decay model using the fitting method of Kemmer and Keller.^43^

### Millisecond Hydrogen–Deuterium Exchange

All HDX experiments were performed at a labeling temperature of 23 °C in triplicate (labeling). The fast-mixing system was directly connected to the digestion/separation chamber of the Waters HDX Manager, with 72 in. long tubing and 75 μm inner diameter, without any insulation. Trapping was performed for 3 min (time to deliver the quenched sample from the sample loop to the pepsin column). Residence in the uninsulated tubing was estimated to be <6 s under a trapping flow rate of 100 μL/min. For the peptide mixture experiments, the pepsin column was replaced with a narrow-bore union.

Labeling was initiated by injecting 20 μL of the peptide mixture or protein into the system by a titrator. During the peptide mixture experiments, the equilibrium buffer syringe contained 20 mM potassium phosphate buffer, pH 7.40 in H_2_O. The labeling buffer, 20 mM potassium phosphate buffer at pH_read_ 7.06 in D_2_O, was placed in the labeling buffer syringe. The mixing ratio in the labeling mixer was 1:20. Depending on the labeling time, this mixture passed through six different loops at different velocities. The labeling reaction was then rapidly quenched by delivering the quench buffer, 100 mM potassium phosphate buffer at pH 2.50 in H_2_O at 0 °C degrees, into the second mixer, which constituted an approximate twofold dilution. The peptide mixture was quenched at 15 time points: 0.05, 0.1, 0.15, 0.20, 0.25, 0.35, 0.5, 0.75, 1, 2.5, 5, 15, 30, 60, and 300 s. The quench buffer was kept in the quench chamber at 0 °C degrees in the quench buffer syringe of the system. GlyPb was analyzed in triplicate in a completely randomized manner at 9 time points including 0.05, 0.15, 0.25, 0.35, 0.5, 1, 5, 30, and 300 s, with the ms2min instrument as described above. Digestion was performed online with a pepsin column. The buffers used while analyzing the protein samples were 40 mM tris hydrochloride and 1 mM TCEP at pH 7.00 in H_2_O, 40 mM tris hydrochloride and 1 mM TCEP at pH_read_ 6.60 in D_2_0, and 100 mM potassium phosphate buffer at pH 2.50 in H_2_O, as carrier, labeling, and quenching buffers, respectively.

### Back-Exchange Correction

Maximally deuterated reference samples^44^ for the peptide mixture were analyzed separately. However, both equilibrium and labeling buffer syringes contained 20 mM potassium phosphate buffer at pH 6.60 in D_2_O. The peptides were dissolved in DMSO-*d*_6_, and all subsequent dilutions prepared in the D_2_O buffer were as described above. The labeling procedure as above was followed, and the labeling was quenched at the same time points as the labeled samples in the ion-exchange experiments.

### Liquid Chromatography–Mass Spectrometry

When required, online digestion, desalting, and separation of the peptides was done on a Waters HDX Manager with an immobilized pepsin column (Enzymate BEH Pepsin Column 2.1 × 30 mm, 5 μm), C18 trapping column (VanGuard ACQUITY BEH 1.7 μm, 2.1 × 5 mm; Waters), and analytical C18 column (1.7 μm, 1.0 × 100 mm ACUITY BEH; Waters). When peptides were analyzed, the pepsin column was removed. Mobile phases were 0.1% formic acid in H_2_O (A) and 0.1% formic acid in ACN (B), such that their pH was 2.50. Peptides were trapped for 4 min at a flow rate of 100 μL/min. Approximately 2.5 pmol of each peptide, or 10 pmol of the protein, was delivered from the fast-mixing system on the column. The peptides were then loaded onto the analytical column and eluted using a linear gradient (15–40% over 4 min at a flow rate of 40 μL/min). During the protein analysis, the separation of the digested peptides was with a linear gradient (5–40% over 7 min at a flow rate of 40 μL/min).

The post-chromatographic separation eluents were directed into a quadrupole time of flight mass analyzer (Synapt G2-Si HDMS QTOF, Waters) with positive ion electrospray ionization tuned for collision-induced dissociation and lock-mass correction (using Leucine enkephalin peptide, 556.2771 *m*/*z*). Mass spectra were obtained in the Waters HDMS^E^ mode (from 50 to 2000 *m*/*z*) for 3D (LC, IM, *m*/*z*) peptide separation. The instrument configuration was as follows: capillary voltage was 3.0 kV, cone voltage was 50 V, trap collision energy was 4 V, and traveling wave ion mobility separation was done with 575 m/s, 36.5 V wave amplitude, and 2.75 mbarN_2_. Transfer collision energy of 4 V was used for low energy scans and four separate ramps between 15 and 55 V for high energy scans.

### Data Analysis

The identity of each peptide was assigned from HDMS^E^ fragment data with ProteinLynx Global Server 3.02 (PLGS) (Waters). Deuterium incorporation was determined with DynamX 3.0 (Waters); the criteria used were: minimum intensity 2000, 2 consecutive products, ppm error 7, and minimum product per amino acid 0.2; all assigned ion spectra were manually curated. The observed uptake was corrected for the observed peptide-specific back exchange, to attain the absolute uptake for each detected peptide. Afterward, the absolute uptake was normalized to the theoretical maximum exchangeable backbone amides (excluding N-terminus and proline) and accounted for approximately 95% D_2_O for each labeling reaction. The deuterium uptake curves were generated by plotting the absolute deuterium uptake (%) against the labeling time. The HDX data are included in the Supporting Information.

### Kinetic Analysis

The intrinsic chemical exchange rate was calculated and simulated for each peptide according to eq 4, where *n* is the number of residues in each peptide, *k*_*i*(int)_ is the intrinsic rate constant of chemical exchange for each residue, and *t* is the labeling time.^33,45−47^
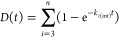
4

At the N-terminus of the peptide, the first amide becomes a primary amine after proteolysis; thus, the first residue back-exchanges quickly during the LC–MS analysis. It has been also reported that the deuterium incorporation on the second residue will also be lost during the separation step.^45,48^ As a result, index *n* starts from the third residue onward. Proline does not contain an amide hydrogen, and so its rate constant will always be zero. The exchange kinetics were quantified as it was described previously,^45^ where the theoretical and experimental deuterium uptake were fitted to a single-, double-, or triple-stretched exponential function.
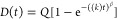
5
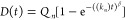
6where *D*(*t*) is the deuterium uptake as a function of the labeling time *t*, *Q* is the number of exchangeable amides, *n* is 3 for max three phases, *k* represents the segment-averaged exchange constant, and β is an exponential stretching factor that accounts for the distribution of the exchange rates of the individual amides. The experimental uptake curves were then fitted in the same manner as the intrinsic curves. The segment averaged protection factors can be estimated by using the ratio of the intrinsic exchange rate constant (*k*_int_) to the measured (experimental) rate constant (*k*_exp_).
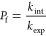
7where *P*_f_ = protection factor against hydrogen exchange, *k*_int_ = intrinsic amide hydrogen exchange rate constant from published values,^45^ and *k*_exp_ = fitted exchange rate constant for back-exchange corrected experimental data.

## Results and Discussion

The newly developed system design termed “ms2min” (to reflect a milliseconds to minutes deuterium-labeling capability; Figure 1) offers fully automated software selection of mixing times over 6 orders of magnitude (milliseconds to minutes) with 1 ms time resolution, labeling temperature control (0–25 °C), quench temperature control (0 °C), online connection for “bottom-up” workflows, two-way communication for reciprocal control of chromatography, automated digestion column wash injection, intercalated blank injections, and sample list scheduling of multiple runs. The notable significant advances over the other designs previously employed are the software selection of mixing times at per millisecond time resolution and the automation of sample list batches.

### “ms2min” Calibration, Timing Accuracy, and Mixing Efficiency

**Figure 2:**
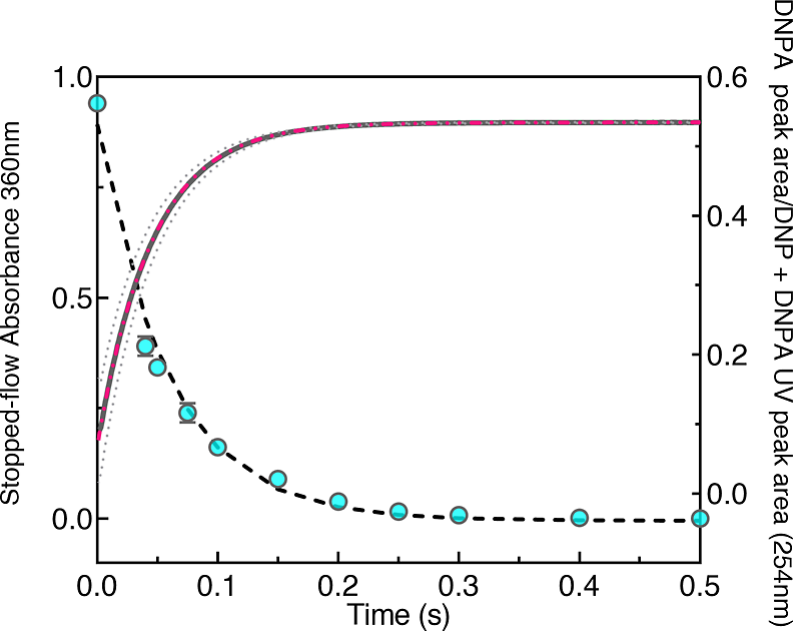
Kinetic data for the hydrolysis of DNPA to DNP in the ms2min prototype (blue circles: error bars are ±2 s.d. from *n* = 3), and SX stopped-flow spectrometer (gray dashed lines are ±2 s.d. from *n* = 9). The data were fitted to a single exponential to provide a first-order rate constant for the ms2min (gray dashed lines) of 20.1 s^–1^ (95% confidence interval range 16.9–23.4 s^–1^) and SX20 (pink line) of 21.85 s^–1^ (95% confidence interval range of 21.62–22.09 s^–1^).

Upon manufacture, each labeling loop on the ms2min system had to be calibrated, in addition to assessing the quality of the mixing. This allows accurate determination of time points, as the combined flow rates delivered by the equilibrium and label buffer syringes and the volume of the selected labeling loop are directly related to the mixing time of labeling. Alkaline catalyzed hydrolysis of DNPA to DNP was used, as it has been proven previously as a powerful control in these types of instruments.^42^ By measuring the kinetics of DNPA hydrolysis with both instruments, we determined the observed rate constants achieved^49,50^ (Figure 2).

The observed rate constants at a hydroxide concentration after mixing of 0.44 M for the ms2min system of 20.1 s^–1^ (95% confidence interval range 16.9–23.4 s^–1^) and SX20 of 21.85 s^–1^ (95% confidence interval range of 21.62–22.09 s^–1^) were in agreement with the second-order rate constant of 48–55 M^–1^ s^–1^ (21.2–24.2 s^–1^ in 0.44 M hydroxide) reported in the literature.^51^

According to the limited scatter of the data and narrow confidence intervals, we conclude that the mixing loops of the ms2min system were accurately calibrated. Furthermore, the relevant velocities to achieve mixing and quenching across the ms-min range were calibrated; thus, the system is suitable for mixing reaction kinetics within the millisecond time regime.

### Per Millisecond Time Resolution

Ideally, H/D measurements are made at precisely the time points that will have the most information content. Therefore, we sought to determine ms2min ability to select and precisely mix any single ms time point (Figure 5B). Ideally, this would offer the freedom of choice of any biologically important time point necessary. We determined the kinetic uptake plots for 5 synthetic peptides from 0.05 to 300 s. These included a 15 ms time window of 1 ms steps in D-labeling time, from 150 to 165 ms (Figure 5B). This range was chosen because it lies on the exponential phase for each of the peptides; therefore, a single millisecond difference in mixing time should have the greatest difference in D-labeling. All 5 kinetic plots were fitted to a stretched exponential model with *R*^2^ ≥ 0.99, excluding the 150–165 ms window, so as not to bias the fitting to the region that would be used to test millisecond precision. Moreover, the data points within the single-millisecond time window (150–165 ms) all lie within the 95% CI of these fits. Therefore, the single millisecond precision of mixing times during the H/D-labeling step appears to be validated. This empowers future experiment design to select whichever D-labeling mixing time is the most appropriate to maximize information content in the data.

### H/D Back-Exchange Is Unaffected by the Mixing Time

**Figure 3:**
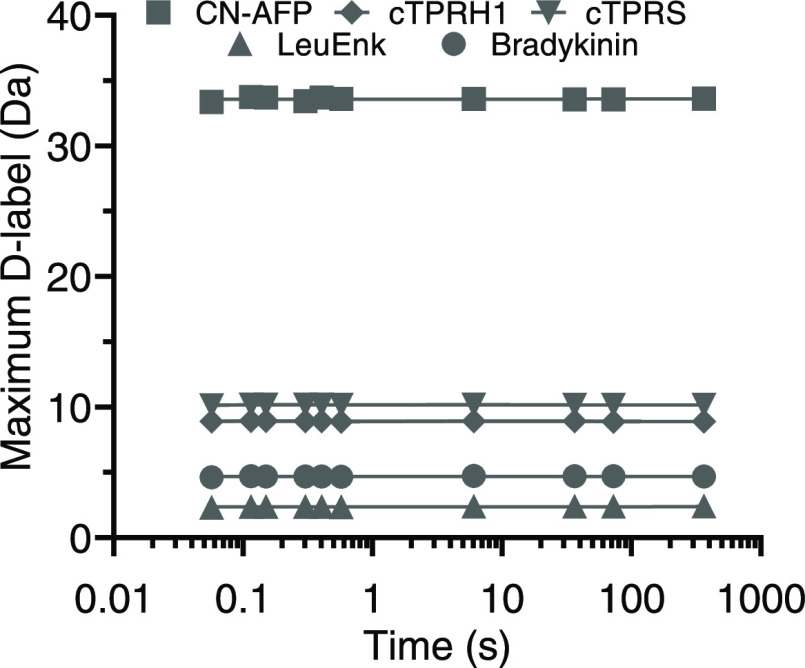
All labeling times tested exhibit an identical back-exchange. The deuterium incorporation observed for fully deuterated synthetic peptides is invariant with the mixing time over more than 5 orders of magnitude. Linear regression fit with gradient <0.00012 in all cases.

Even though much effort is made to minimize back-exchange, it is still prevalent in HDX-MS experiments. In the ms2min system, for different mixing (D-labeling) times, the sample experiences different flow velocities and characteristics. This has the potential to result in variation in back-exchange, which would confound both (i) the aim to acquire accurate D-labeling kinetics and (ii) attempts to correct for the loss of deuterium (back-exchange), which is required to compare proteoforms and to calculate the free energy of stability values. Therefore, we confirmed that there is no back-exchange variation with mixing time by analyzing five fully deuterated peptides with the ms2min system. The highest measured dependence of back exchange on mixing time is 0.012% over the 300 s time range tested, given by linear regression (Figure 3). In all five peptides, for labeling across 4 orders of magnitude of time, no significant correlation could be identified as indicated by the poor correlation coefficient to a linear regression model (*R*^2^ ≤ 0.32). Therefore, we were able to correct for back exchange within an experiment by reference to a single fully deuterated sample.

We additionally compared the loss of deuterium during ms2min analysis to the loss experienced with conventional CTC PAL-based robotics systems. The five peptides were tested in the same exact manner, after each being dissolved in 99.9% D_2_O buffer. Note that following quenching, the LC–MS regimes are identical for both systems. These are compared in Table S2. Interestingly, bradykinin and CN-AFP showed 50% more deuterium retained with the ms2min system, while the other peptides had insignificant differences between the instruments.

### Millisecond Kinetics of Unstructured Peptides

Peptides often have weak protection factors against amide hydrogen exchange.^45,46^ This renders them intractable to measure by conventional sampling as most of the labeling is complete within the dead time of the experiment. We determined the lower limit to which the ms2min system can quantify the peptide stability in solution by measuring five peptides—a hormone (bradykinin), a neurotransmitter (leucine enkephalin), a consensus sequence antifreeze protein (CN-AFP), and in-lab synthesized peptides: cTPR1 and cTPRS (Figure 5A). The deuterium incorporation was measured at 15 time points ranging from 0.05 to 300 s and was compared with their calculated maximum intrinsic exchange rates (i.e., rate corresponding to a fully unprotected species^45^).

The data were corrected for back exchange, permitting direct comparison between the measured and theoretical chemical HDX rates. As the observed deuterium incorporation is the sum of a number (*q*—corresponding to the exchangeable amide groups in the peptide) of exponential exchange rates (*k*_j_), the ideal case would be to fit the data to *q* exponential phases. This becomes overly complex for *q* > 3, given the number of time points that would be needed to be sampled. Therefore, we fit the data to a stretched exponential function with the least number of phases required to generate a good fit. We used this ensemble rate constant to calculate per peptide protection factors (*P*_f_). We were able to generate good fits to the data and obtain *P*_f_ values for each peptide (Tables 1 and S1). Interestingly, bradykinin, cTPRH1, and cTPRS were observed to be natively disordered in the solution, with no protection against maximum theoretical hydrogen exchange rates (Figure 5A). Some of the peptides showed faster kinetics than the theoretically calculated ones. It was demonstrated previously that intrinsic rate constant calculation can potentially underestimate the deuterium incorporation.^52^

**Table 1:** Calculated Peptide Averaged Protection Factors

	ln (*P*_f_)		
bradykinin	–0.14	–0.42	
CN-AFP	1.83		
LeuEnk	1.79	0.86	–0.15
cTPRH1	0.28	–2.15	
cTPRS	–0.21	–4.24	

The gray shaded region in Figure 5A denotes the conventional HDX time frame. In each case, conventional HDX-MS labeling would be too slow to measure peptide dynamics and stability. All five peptides contain a single amide proton that exchanges even faster than the dead time of this experiment (50 ms), indicating that there is additional information to be gained from faster instrument performance in future.

### Validation of Data Reproducibility

**Figure 4:**
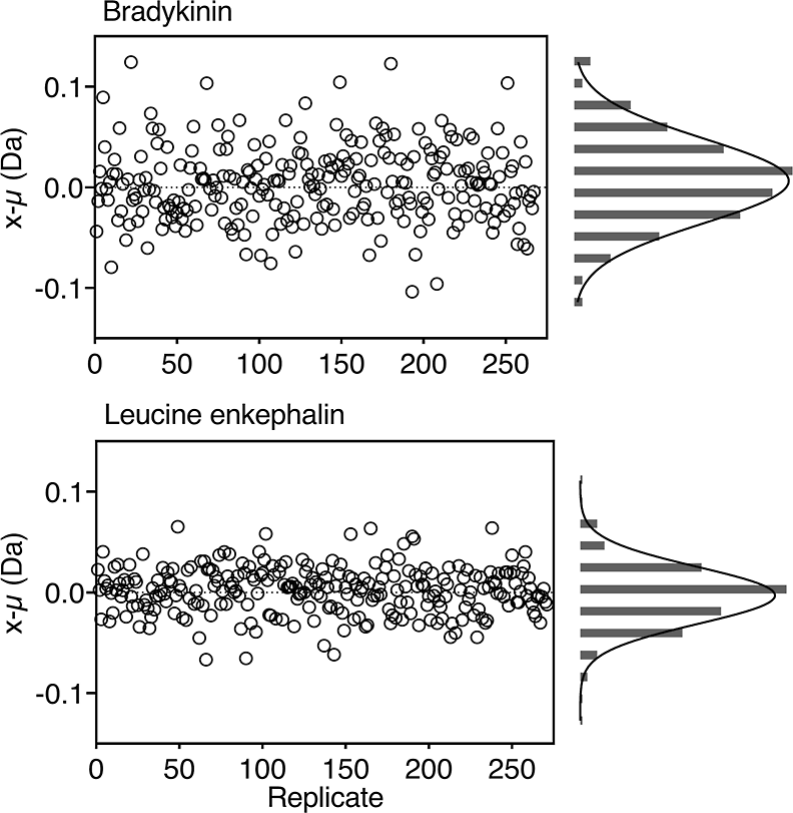
Deviation from the mean measurement of deuterium label at 100 ms for bradykinin and leucine enkephalin; *n* = 270 replicate data points with a dispersion of 3%.

To further validate the ms2min system, we determined the measurement repeatability/reproducibility and labeling precision. On three separate days, we measured the deuterium uptake of 50, 70, and 150 technical replicates of bradykinin and leucine enkephalin peptides at a mixing time of 100 ms (Figure 4). The short labeling period close to the limit of quantification was selected as these data points can be very sensitive to variability in the labeling reaction. Random, uniform, and symmetrical distribution of the deviation from the mean (μ) was observed with <0.01 Da variability between days (Figure 4). The 95% confidence interval was ±0.01 Da (0.2% of the signal amplitude) for both bradykinin and leucine enkephalin. This further confirmed that the calibration and design of the system was suitable for ms2min labeling measurements.

**Figure 5:**
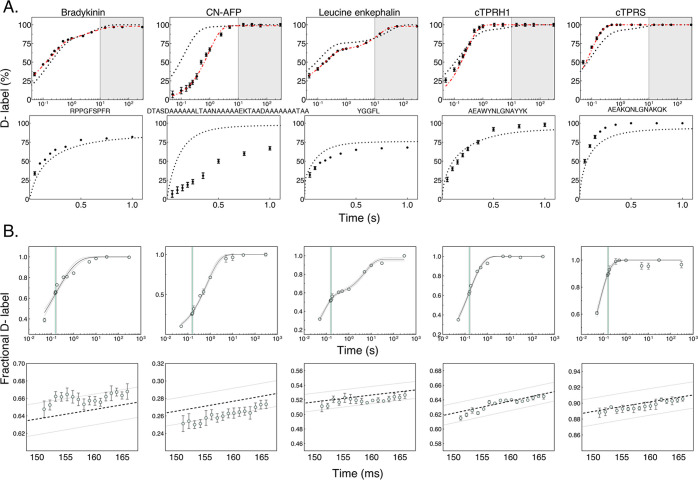
(A) Synthetic peptide exchange at different rates depending on their structure. Absolute hydrogen exchange (expressed as % deuteration) shown for five peptides compared with the theoretical maximum chemical exchange (black dotted curve); error bars denote ±1 r.s.d. from *n* = 3. Stretched exponential fit to experimental data (red dashed line). Plots showing the full time course (top) and succinct time course up to 1 s (bottom). The gray shaded region denotes the time achieved by most conventional HDX systems. (B) Kinetic plots of relative deuterium uptake (Da) for five peptides from 50 ms to 300 s (top); error bars denote ±1 r.s.d. from *n* = 3. The blue shaded region represents the window tested for a single millisecond time resolution from 150 to 165 ms, with an expanded view (bottom), ±1 r.s.d. from *n* = 3.

### ms2min of Apo (T-form) GlyPb Dimer

**Figure 6:**
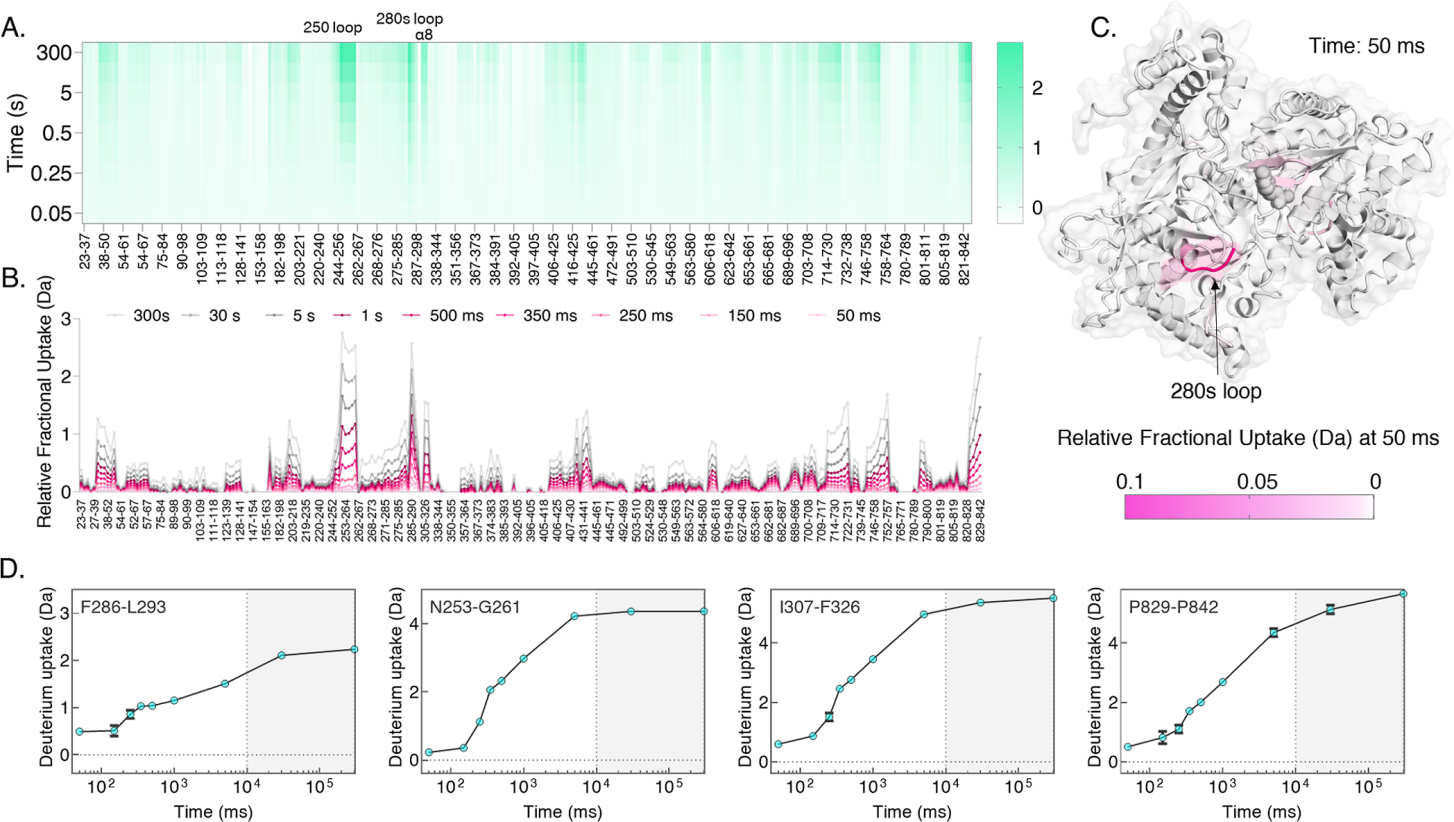
GlyPb labeling map with the ms2min system. (A) Heat map of deuterium uptake per peptide segment, at 9 time points from 50 ms to 300 s, indicates the extent of the hydrogen exchange at each labeling time (rows), given as relative fractional uptake of deuterium (%). (B) Butterfly plot showing the relative fractional uptake per time point (pink lines are the time points from 50 ms to 1 s). Horizontal scale denotes the peptide segments seen during the analysis. (C) Overlaid uptake data on PDB:1NOI for 50 ms. (D) Deuterium uptake plots (*n* = 3) for several significant segments including F286-L293, N253-G261, I307-F326, and P829-P842.

We sought to assess the ms2min system by analyzing a large molecule in a completely automated fashion. GlyPb is an allosteric enzyme that forms a dimer of a large (∼195 kDa) polypeptide chain. As such, it represents a challenging and widely used molecule to indicate HDX-MS system performance and sensitivity. As previously explained, GlyPb was dissolved in 40 mM tris hydrochloride and 1 mM TCEP at pH 7.00 to a final concentration of 10 μM. A full time course was set up on the ms2min system from 50 ms to 300 s, *n* = 3, in a randomized schedule. The reaction was initiated by introducing 20 μL of GlyPb onto the ms2min system. The sample consumption was extensive and has been improved upon separately.^53^ Mass spectral assignment of GlyPb peptides resulting from the pepsin enzyme digest provided the identification of 273 peptides, corresponding to 96.4% protein coverage (Figure S1). The full time course resulted in high temporal and structural resolution data (Figure 6A,B), providing excellent information about solution phase local stability and structural perturbation of GlyPb. As GlyPb is a dimer with a highly structured and rigid core, HDX uptake on the millisecond scale was only observed in highly unstructured regions. These include the 280 s loop, the 250s′ loop, α8, and the C-terminal (Figure 6D).

To further estimate the advances achieved with the ms2min system, we compared the information content available from the conventional (≥seconds) and the millisecond time range (Figure 6D) (the gray shaded region denotes the conventionally acquired time window data). Data from the ms2min in the 1–300 s time domain were similar to published values from CTC-PAL experiments (given limitations in the interpretation from variation in the labeling conditions and LC–MS systems).^54^ We found certain regions in this large enzyme that have little to no information content by HDX-MS when resolved at the seconds-minutes time window, but almost complete kinetics were obtained when the HDX window was extended by 3 orders of magnitude, down to 50 ms. Insights into the structural changes upon Ser14 phosphorylation were attained, though it is evident that expansion of the time window stands to detect structural perturbations more sensitively.

The structural perturbations observed with HDX correlate highly with previous studies and crystal structures. As evident, the 250′ (253–261) and 280 s (286–293) loops both engaged in the activation of GlyPb are highly unstructured and almost undetectable in the conventional HDX window. Even at 50 ms (Figure 6C), the relative fractional uptake (%) of these regions can be unambiguously observed. As the 280 s loop is considered the active site gate, acquiring data in the millisecond range is crucial for studying the structural perturbation upon allosteric activation.

## Conclusions

In this study, the design, development, and validation of the “ms2min” system was described, and its efficiency in performing millisecond HDX mixing was demonstrated. Our initial tests on synthetic peptide hormone (bradykinin), neurotransmitter (leucine enkephalin), consensus anti-freeze protein (CN-AFP), and in-lab synthesized peptides, cTPR1 and cTPRS, validated the approach by permitting the calculation of protection factors, even in natively unstructured regions. The HDX uptake map of GlyPb produced with the “ms2min” system was comparable to conventional sample handling procedures. The reproducible millisecond time points aided and expanded the window, allowing easier calculation of stability in unstructured regions in large proteins.

The reproducibility of the mixing at 100 ms showed that random uniform distribution was observed with <0.01 Da variability between days. The back exchange experienced during mixing (passing through different labeling loops) was also determined. The poor correlation coefficient to a linear regression model (*R*^2^ ≤ 0.32) demonstrated that no significant correlation could be identified, between all six mixing loops. This ensures that HDX forward and backward exchange occur as expected with the ms2min system, as is the case for manual or CTC/PAL-based experiments.

The new “ms2min” system developed here has significant merits from a technical standpoint: considerable potential for HDX-MS resolved at the millisecond level has been demonstrated previously, for example, applied to the study of protein folding.^2^ The ms2min system stands to be particularly impactful in the future study of intrinsically disordered proteins/regions^53,55^ and for emerging time-resolved (i.e., non-equilibrium) protein conformational dynamics, for example, applied to transmission electron microscopy lactamase catalysis,^5^ given its software control over single millisecond differences in the labeling time and online full automation. Given a recently proposed framework for temperature correction to quantitatively expand the time window of HDX-MS measurements, the ms2min instrument should be capable of sub-millisecond timepoints at a reference temperature of 298 K.^56^ The recent development of sub-zero LC for bottom-up HDX-MS will expand the technique to vastly more complicated samples, such as blood plasma, crude cell lysate, and cell culture media. We envisage that coupling these chromatographic advances to millisecond HDX pulse labeling will yield new information such as weak binding interactions (that have been shown to be detectable by TRESI HDX-MS) in/ex vivo.^57^

## Supporting Information Available

GlyPb coverage map; parameters obtained from manual fitting of theoretical and experimental data acquired for bradykinin, CN-AFP, leucine enkephalin, cTPRH1, and cTPRS; comparison of the back exchange observed when using the CTC-PAL automation and the ms2min system; GlyPb HDX MS source data; and HDX data peptides (PDF)
