# Supplementary material for: Online Fully Automated System for Hydrogen/Deuterium-Exchange Mass Spectrometry with Millisecond Time Resolution
Source: Anal Chem. 2023 Mar 10;95(11):5000–8. doi: 10.1021/acs.analchem.2c05310 (PMC10034745; doi:10.1021/acs.analchem.2c05310)
Supplement: Supplementary file 1 — ac2c05310_si_001.pdf [file ac2c05310_si_001.pdf]

Supporting information

An online fully automated system for hydrogen/ deuterium-exchange mass spectrometry with millisecond time resolution

Monika Kish[1], Victoria Smith[2], Natasha Lethbridge[2], Lindsay Cole[3], Nicholas. J. Bond[4] and Jonathan J. Phillips[*1,5]

[1]Living Systems Institute, Department of Biosciences, University of Exeter, Stocker Road, Exeter, EX4 4QD, UK

[2]CPI, Darlington, DL1 1GL, UK

[3]Applied Photophysics Ltd, Leatherhead, KT227BA, UK

[4]Analytical Sciences, Biopharmaceutical Development, BioPharmaceuticals R&D, AstraZeneca, Milstein Building, Granta Park, Cambridge, CB21 6GH, UK

[5]Alan Turing Institute, British Library, London, NW1 2DB, UK

*Correspondence to: jj.phillips@exeter.ac.uk

Table of contents:

Table S1 Parameters obtained from manual fitting of theoretical and experimental data acquired for Bradykinin, CN-AFP, Leucine Enkephalin, cTPRH1 and cTPRS. 1
Table S2 Comparison of the back exchange observed when using the CTC-PAL automation and the ms2min system... 2

Figure S1. Sequence coverage of glycogen phosphorylase b from rabbit muscle.. 2
Figure S2. Qualitative comparison of published HDX-MS data for GlyPb generated using CTC-PAL labeling instrument 2

Table S1 Parameters obtained from manual fitting of theoretical and experimental data acquired for Bradykinin, CN-AFP, Leucine Enkephalin, cTPRH1 and cTPRS.

Peptide	Measured							Intrinsic						
	N_1	N_2	N_3	k_1	k_2	k_3	β	N_1	N_2	N_3	k_1	k_2	k_3	β
				(s^{-1})	(s^{-1})	(s^{-1})					(s^{-1})	(s^{-1})	(s^{-1})	
Bradykinin	1.10	3.80		0.21	8.39		0.68	1.18	3.83		0.18	5.49		0.81
CN-AFP	35.54			1.15			0.96	35.66			7.16			0.84
Leucine Enkephalin	1.00	1.01	1.98	50.43	0.04	3.24	1	1.01	1.00	1.99	0.03	302.60	7.61	0.98
cTPRH1	10.90	1.16		5.08	1.00		0.94	10.88	1.13		6.75	0.12		0.67
cTPRS	10.83	1.17		12.78	12.80		0.86	10.83	1.17		10.38	0.18		0.71

Table S2 Comparison of the back exchange observed when using the CTC-PAL automation and the ms2min system. The back exchange shows a similar trend between the peptides analyzed by CTC-PAL automation, though the ms2min system preserves more of the deuterium label.

	Maximum number of deuterons	Back-exchange CTC-PAL (LEAP)	Back-exchange ms2min
Bradykinin	5	15%	6%
CN-AFP	36	13%	7%
LeuEnk	4	49%	41%
cTPRH1	12	14%	15%
cTPRS	12	29%	26%

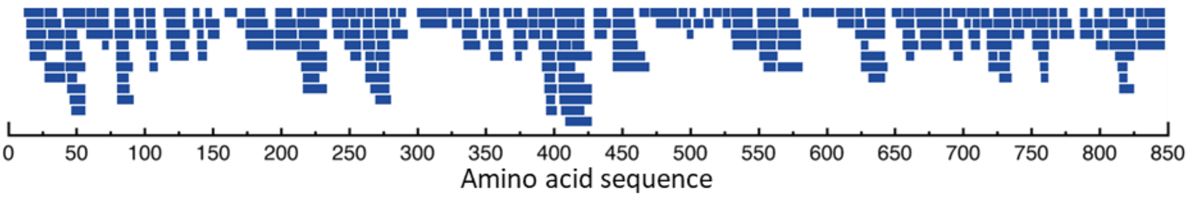

Figure S1. Sequence coverage of glycogen phosphorylase b from rabbit muscle. The peptides obtained via peptic digestion and LC-IMS/MS analysis are shown as blue bars along the sequence numbers. Each bar under the sequence number annotation indicates an identified peptic peptide that was monitored during all HDX-MS experiments. These 273 peptides cover up to 800 amino acids of the total amino acid residues in the proteins, yielding a linear sequence coverage of up to 94.9%, with 4.04 redundancy.

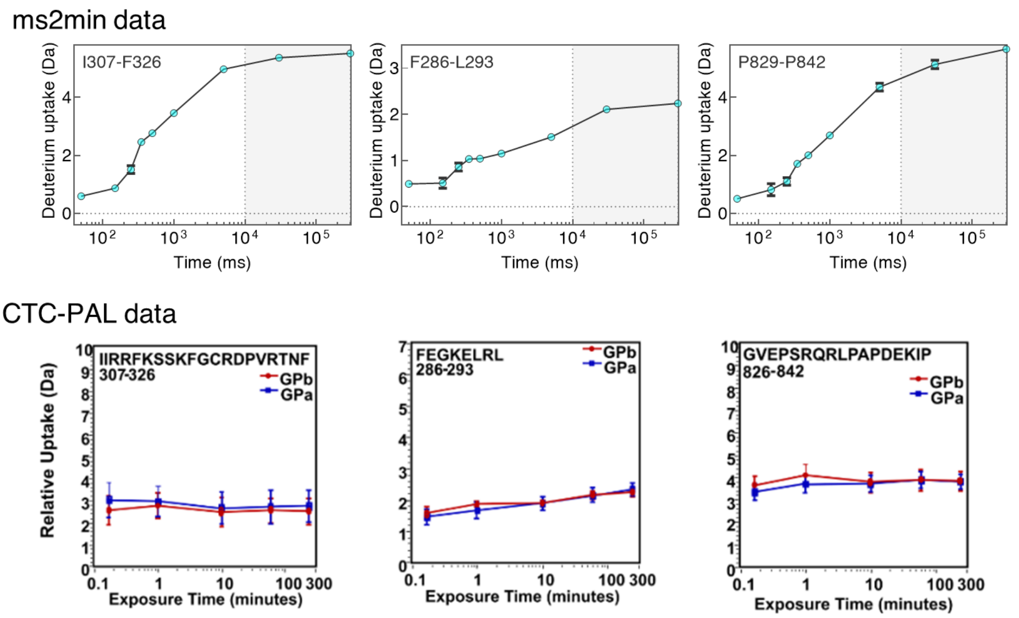

Figure S2**.** Qualitative comparison of published HDX-MS data for GlyPb generated using CTC-PAL labeling instrument (red trace in lower panels) and data generated using ms2min instrument (upper panels). Data reproduced from Huang, J.; Chu, X.; Luo, Y.; Wang, Y.; Zhang, Y.; Zhang, Y.; Li, H. ACS Chem Biol **2022**, 17 (7), 1951-1962.

fa
